# Methodological insights into ChatGPT’s screening performance in systematic reviews

**DOI:** 10.1186/s12874-024-02203-8

**Published:** 2024-03-27

**Authors:** Mahbod Issaiy, Hossein Ghanaati, Shahriar Kolahi, Madjid Shakiba, Amir Hossein Jalali, Diana Zarei, Sina Kazemian, Mahsa Alborzi Avanaki, Kavous Firouznia

**Affiliations:** 1 Advanced Diagnostic and Interventional Radiology Research Center (ADIR), Tehran University of Medical Science, Tehran, Iran; 2 Cardiac Primary Prevention Research Center, Cardiovascular Diseases Research Institute, Tehran University of Medical Sciences, Tehran, Iran

**Keywords:** Systematic review, ChatGPT, AI, Large language model, Article screening, Radiology, GPT

## Abstract

**Background:**

The screening process for systematic reviews and meta-analyses in medical research is a labor-intensive and time-consuming task. While machine learning and deep learning have been applied to facilitate this process, these methods often require training data and user annotation. This study aims to assess the efficacy of ChatGPT, a large language model based on the Generative Pretrained Transformers (GPT) architecture, in automating the screening process for systematic reviews in radiology without the need for training data.

**Methods:**

A prospective simulation study was conducted between May 2nd and 24th, 2023, comparing ChatGPT’s performance in screening abstracts against that of general physicians (GPs). A total of 1198 abstracts across three subfields of radiology were evaluated. Metrics such as sensitivity, specificity, positive and negative predictive values (PPV and NPV), workload saving, and others were employed. Statistical analyses included the Kappa coefficient for inter-rater agreement, ROC curve plotting, AUC calculation, and bootstrapping for p-values and confidence intervals.

**Results:**

ChatGPT completed the screening process within an hour, while GPs took an average of 7–10 days. The AI model achieved a sensitivity of 95% and an NPV of 99%, slightly outperforming the GPs’ sensitive consensus (i.e., including records if at least one person includes them). It also exhibited remarkably low false negative counts and high workload savings, ranging from 40 to 83%. However, ChatGPT had lower specificity and PPV compared to human raters. The average Kappa agreement between ChatGPT and other raters was 0.27.

**Conclusions:**

ChatGPT shows promise in automating the article screening phase of systematic reviews, achieving high sensitivity and workload savings. While not entirely replacing human expertise, it could serve as an efficient first-line screening tool, particularly in reducing the burden on human resources. Further studies are needed to fine-tune its capabilities and validate its utility across different medical subfields.

**Supplementary Information:**

The online version contains supplementary material available at 10.1186/s12874-024-02203-8.

## Introduction

The domain of deep learning has witnessed significant development over the past decade, dramatically transforming numerous fields, including medicine [1]. Machine translation, an application of deep learning that employs computer algorithms to automatically translate text or speech from one language to another, has achieved remarkable advancements in recent years. The advent of Attention mechanisms and the subsequent introduction of the Transformer architecture, proposing the self-attention concept, has revolutionized this field [2, 3].

The Transformer architecture forms the backbone of many large language models (LLMs). It uses a certain type of neural network that comprises two components—an encoder and a decoder. The encoder analyzes the input, which is a sequence of words, helping the network understand the overall context. While the decoder generates an output sequence conditioned on the established context. However, not all neural network models use both components. Some only utilize the encoder part (e.g., Bidirectional Encoder Representations from Transformers [BERT]), some the decoder part (e.g., Generative Pretrained Transformer [GPT]), and some both (e.g., Text-to-Text Transfer Transformer [T5]) [4–6].

GPT models, which utilize the decoder portion of the Transformer architecture, have achieved considerable success in text completion tasks [5, 7]. The recent development of ChatGPT, an LLM primarily based on GPT-3.5 and reinforced with human feedback (RLHF), extends its capabilities beyond text completion [8]. It can answer questions, maintain human-like dialogue, provide assistance, devise plans, and write performant code [9].

In the medical literature, systematic reviews and meta-analyses occupy the apex of the evidence pyramid [10]. They collect, critically appraise, and synthesize the results of multiple studies within a specific field. The production of these types of studies demands considerable effort due to the numerous steps required to ensure fair and comprehensive results. One of the earliest steps, the article screening phase, can be particularly labor-intensive and time-consuming. However, since it controls what studies are fed into the process, it is of utmost importance. It is vital that the systematic review’s conclusions are drawn based on the best available evidence, free from bias, and relevant to the research question. Errors at this stage can severely degrade the review’s validity and its utility in guiding practice and policy.

Historically, several studies have sought to apply machine learning or deep learning methods to assist in this process [11–14]. Despite these efforts, they usually require some form of annotation input by the user and mostly have evaluated their performance on retrospective data. Our study seeks to automate the process without the need for training. We hypothesize that delegating a portion of manual labor to ChatGPT can reduce missed potential articles and increase efficiency while conserving human resources. To scrutinize our hypothesis, we set up this study to assess the efficacy of ChatGPT concerning the screening process and compared its performance to human raters.

## Materials and methods

### Study design

We undertook a prospective simulation study from May 2nd to 24th 2023, designed to assess the accuracy and speed of ChatGPT in screening abstracts for systematic reviews. Our main objective was to measure how effective ChatGPT is in reliably excluding abstracts collected from the primary screened results of a systematic review. We also compared ChatGPT against a group of researchers, specifically general physicians (GPs), who are typically involved in the abstract screening process.

We used metrics such as sensitivity, specificity, precision or positive predictive value (PPV), negative predictive value (NPV), positive likelihood ratio (PLR), negative likelihood ratio (NLR), false negative rate, proportion missed, and workload saving to evaluate performance. False negative rate (FNR) is the proportion of actual positive cases that were incorrectly classified as negative. The proportion missed is similar to the FNR but is often expressed in a different context. It is the number of relevant studies that the rater has failed to identify, out of those it predicted to be irrelevant. This is essentially a measure of how many relevant studies were missed by the rater. Workload saving is the proportion of citations that were correctly identified as irrelevant, thereby reducing the workload for human reviewers. It is the proportion of citations predicted irrelevant out of the total number of citations. Below is the mathematical description for them:

$$FNR=\frac{FN}{TP+FN}, \,\,Proportion \,Missed= \frac{FN}{FN+TN}, \,\,Workload \,Saving= \frac{TN}{TN+FN+TP+FP}$$

FN: False negative, TP: True positive, TN: True negative, FP: False positive.

### Data collection

We surveyed three extensive fields of radiology: diagnostic, interventional, and nuclear medicine. Six synthetic broad topics were proportionally proposed based on the distribution of corresponding PubMed search results frequency, then we designed a PICOS (Population, Intervention, Comparison, Outcomes, Study design) for each topic (Additional file 1: Table S1). It is noteworthy that the topics and their corresponding PICOS were conceived by our group of experts focusing on broad and diverse subjects. Subsequently, we systematically searched PubMed, Embase, and Web of Science from inception until April 29th, 2023 (Additional file 1: Table S2) using the queries that were carefully designed by the author and verified by the experts. We aimed for broader rather than specific terms when choosing the keywords as one would for real scenarios. Thereafter, we eliminated duplicates and citations that were missing abstracts using the software package EndNote X9 [15]. Next, due to the constraints of time and human resources, a random subset of 200 articles from each topic was selected using a Python script.

Three general physicians, who had experience in medical research synthesis and systematic review composition for over a year, were independently given the topics, corresponding PICOS, titles, and abstracts to screen. They were unaware that they were being compared to other raters and AI. Moreover, raters were not allowed to access the full texts of the articles. Their task was to determine whether to include or exclude the citation based solely on the provided PICOS, the title, and the abstract. Each individual marked 1198 citations (~ 200 articles for each of the six topics) as either included or excluded. Two experts, including a physician with over twenty years of research experience in radiology and a faculty member radiologist with more than five years of research experience in the field, both having previously published systematic reviews, were assigned the same task. The study employed a fair and thorough process for resolving any disagreements between the two experts. In such cases, a third expert—a physician with over two decades of research experience and published systematic reviews in the field—was consulted. The third expert was unaware of the identities of the previous experts and made the final decision in such situations. The final verdicts of the expert group were considered the study’s ground truth.

Overall, three types of consensuses were employed for each of the GP and expert groups: sensitive consensus included studies if at least one of the raters included them, specific consensus included studies if all of the raters included them, and voting consensus included studies if the majority of raters included them (in the case of the experts’ group, denoting the verdict of the third expert). Thus, in total, we had 6 outputs from the GPs group and 3 outputs from the experts group.

Finally, we interfaced ChatGPT via a custom Python script and OpenAI’s application programming interface (API), prompting it to rate the citations on a scale of 1 (least relevance) to 5 (most relevance) based on the provided PICOS. We only presented ChatGPT with titles, abstracts, reference types, publish dates and PICOS. This study utilized the May 3rd release of ChatGPT (specifically, “gpt-3.5-turbo”) with the parameter “temperature” set to 0.0 for a more deterministic behavior or “greedy search”.

### Statistical analysis

Data preprocessing, cleaning, and analysis were accomplished with Python version 3.9.13, supported by various libraries such as *pandas* (for data-frame manipulation), *numpy* (for math and random number generation), *random* (for random sampling of the articles), *scikit-learn* (for statistical tests and metrics), and *matplotlib* and *seaborn* (for plotting) [16–20]. Our analysis included the computation of the Kappa (κ) coefficient for inter-rater agreement, plotting receiver operating characteristic curve (ROC), calculation of the area under the curve (AUC) or c-statistic, Youden’s index and threshold, and various other metrics, including sensitivity, specificity, PPV, NPV, PLR, NLR, FNR, proportion missed, workload saving, Jaccard index or Intersection over Union (IoU), and balanced accuracy [21–24]. Balanced accuracy was used to account for the imbalance present in our data. Confidence intervals (CIs) were calculated with a 95% threshold, and p-values below 0.05 were considered statistically significant. For the calculation of p-values, confidence intervals, and comparison between the metrics, bootstrapping with 1000 samples was employed [25]. Numbers in square brackets ([]) denote confidence interval.

## Results

**Fig. 1:**
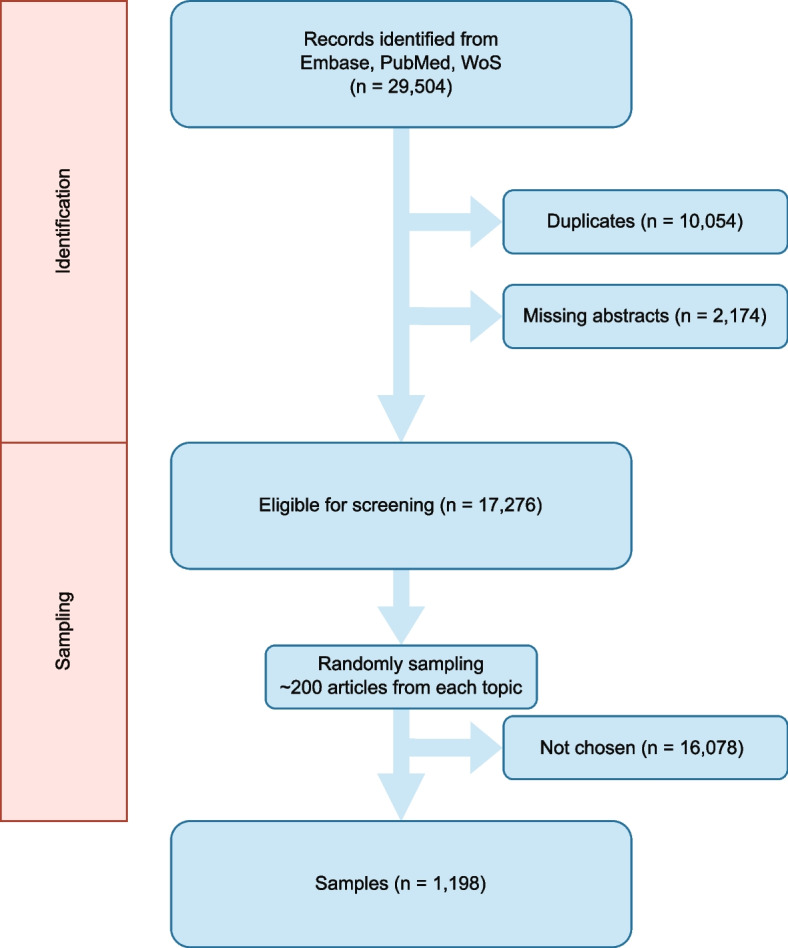
Article identification and sampling process. WoS: Web of Science

Overall, the study involved the review of 1,198 abstracts and titles (Fig. 1, Table 1). The topics were chosen conforming to the following distribution: 3/6, 2/6, and 1/6 concerning diagnostic radiology, nuclear medicine, and interventional radiology respectively. Further details regarding the systematic searches for each topic are presented in Additional file 1: Table S2.

**Table 1:** Selected topics and corresponding sampling details

Field	Title (topic)	Total found	Duplicates	Missing abstracts	Final	Sample proportion
Diagnostic Radiology	The diagnostic accuracy of CT colonography in detecting colorectal polyps and cancer (COLORECTAL)	3263	1564	338	1361	14.7%
	Computed tomography angiography versus magnetic resonance angiography for the diagnosis of peripheral arterial disease (PAD)	307	74	35	198	100.0%
	Accuracy of ultrasound in diagnosing deep vein thrombosis (DVT)	21,802	7164	1509	13,129	1.5%
Nuclear Medicine	Diagnostic accuracy of PET imaging with 18F-FDG in differentiating malignant from benign adrenal tumors (PET)	831	234	25	572	35.0%
	Diagnostic performance of SPECT and SPECT/CT in detecting bone metastases (SPECT)	2533	812	198	1523	13.1%
Interventional Radiology	Comparative effectiveness of drug-eluting stents versus bare metal stents in patients with peripheral artery disease (STENT)	768	206	69	493	40.6%

Three general physicians and two experts independently assessed the citations for inclusion. The review process took the GPs 7, 8, and 10 days (averaging ~ 2–3 h of work per day) (Additional file 1: Table S3), while it took the experts 3 and 5 days to rate and one day for the third expert to reach final verdicts (55 disagreements were addressed in total, Additional file 1: Table S4). In contrast, ChatGPT completed the process in less than one hour (~ 3 s per citation or ~ 1 h in total).

For the sake of brevity, we only considered the voting consensus (the final verdict reached by the third expert) as the gold standard in this study. More detailed results including alternative gold standards are presented in Additional file 1: Figures S2 to S9.

### Inter-rater agreement

The average inter-rater agreement, as measured by the kappa statistic [κ], was moderate among the GPs at 0.45, and substantial between the two experts at 0.79. However, the agreement between ChatGPT, at threshold ≥ 3, and the other raters was lower with a mean kappa of 0.27. Details are provided in Table 2.

**Table 2:** Inter-rater agreements

Rater	Versus	Kappa (κ)	95% CI
GP 1	GP 2	0.47	0.39–0.55
	GP 3	0.38	0.31–0.45
	Expert 1	0.53	0.45–0.60
	Expert 2	0.48	0.40–0.55
	ChatGPT	0.28	0.24–0.33
GP 2	GP 3	0.51	0.43–0.59
	Expert 1	0.60	0.52–0.67
	Expert 2	0.57	0.49–0.65
	ChatGPT	0.20	0.16–0.23
GP 3	Expert 1	0.66	0.59–0.72
	Expert 2	0.59	0.52–0.65
	ChatGPT	0.30	0.25–0.34
Expert 1	Expert 2	0.79	0.73–0.84
	ChatGPT	0.29	0.25–0.34
Expert 2	ChatGPT	0.28	0.24–0.33

### Screening performance

Comparing GP consensuses—sensitive, specific, and voting—with our gold standard, they achieved sensitivities of 90%, 32%, and 62%, respectively (Table 3). As evident in the table, sensitive consensus performs better in almost every aspect compared to each individual.

**Table 3:** Comparing human raters against ChatGPT at threshold ≥ 3, across the whole dataset

Human raters	Evaluation METRIC	Value [95% CI]	ChatGPT [95% CI]	P-value (two-tailed)
GP 1	Sensitivity	0.55 [0.48,0.63]	0.95 [0.91,0.98]	< 0.001
	Specificity	0.94 [0.93,0.96]	0.65 [0.62,0.68]	< 0.001
	Precision (PPV)	0.58 [0.50,0.66]	0.28 [0.24,0.32]	< 0.001
	Negative Predictive Value	0.94 [0.92,0.95]	0.99 [0.98,1.00]	< 0.001
	Positive Likelihood Ratio	9.70 [7.48,13.34]	2.71 [2.46,3.01]	< 0.001
	Negative Likelihood Ratio	0.47 [0.39,0.56]	0.08 [0.03,0.14]	< 0.001
	Balanced Accuracy	0.75 [0.71,0.79]	0.80 [0.77,0.82]	0.016
	Jaccard Index	0.39 [0.33,0.47]	0.27 [0.23,0.31]	< 0.001
	False Negative Rate	0.45 [0.37,0.52]	0.05 [0.02,0.09]	< 0.001
	Proportion Missed	0.06 [0.05,0.08]	0.01 [0.00,0.02]	< 0.001
GP 2	Sensitivity	0.55 [0.46,0.63]	0.95 [0.91,0.98]	< 0.001
	Specificity	0.99 [0.98,0.99]	0.65 [0.62,0.68]	< 0.001
	Precision (PPV)	0.86 [0.79,0.93]	0.28 [0.24,0.32]	< 0.001
	Negative Predictive Value	0.94 [0.92,0.95]	0.99 [0.98,1.00]	< 0.001
	Positive Likelihood Ratio	44.20 [26.11,90.48]	2.71 [2.46,3.01]	< 0.001
	Negative Likelihood Ratio	0.46 [0.38,0.54]	0.08 [0.03,0.14]	< 0.001
	Balanced Accuracy	0.77 [0.72,0.81]	0.80 [0.77,0.82]	0.16
	Jaccard Index	0.50 [0.42,0.58]	0.27 [0.23,0.31]	< 0.001
	False Negative Rate	0.45 [0.37,0.54]	0.05 [0.02,0.09]	< 0.001
	Proportion Missed	0.06 [0.05,0.08]	0.01 [0.00,0.02]	< 0.001
GP 3	Sensitivity	0.74 [0.66,0.80]	0.95 [0.91,0.98]	< 0.001
	Specificity	0.94 [0.92,0.95]	0.65 [0.62,0.68]	< 0.001
	Precision (PPV)	0.62 [0.55,0.68]	0.28 [0.24,0.32]	< 0.001
	Negative Predictive Value	0.96 [0.95,0.97]	0.99 [0.98,1.00]	< 0.001
	Positive Likelihood Ratio	11.37 [9.00,14.60]	2.71 [2.46,3.01]	< 0.001
	Negative Likelihood Ratio	0.28 [0.21,0.36]	0.08 [0.03,0.14]	< 0.001
	Balanced Accuracy	0.84 [0.80,0.87]	0.80 [0.77,0.82]	0.076
	Jaccard Index	0.50 [0.44,0.57]	0.27 [0.23,0.31]	< 0.001
	False Negative Rate	0.26 [0.20,0.34]	0.05 [0.02,0.09]	< 0.001
	Proportion Missed	0.04 [0.03,0.05]	0.01 [0.00,0.02]	< 0.001
Voting Consensus (GPs)	Sensitivity	0.62 [0.54,0.70]	0.95 [0.91,0.98]	< 0.001
	Specificity	0.98 [0.97,0.99]	0.65 [0.62,0.68]	< 0.001
	Precision (PPV)	0.83 [0.75,0.89]	0.28 [0.24,0.32]	< 0.001
	Negative Predictive Value	0.95 [0.93,0.96]	0.99 [0.98,1.00]	< 0.001
	Positive Likelihood Ratio	34.35 [22.94,58.37]	2.71 [2.46,3.01]	< 0.001
	Negative Likelihood Ratio	0.39 [0.31,0.46]	0.08 [0.03,0.14]	< 0.001
	Balanced Accuracy	0.80 [0.76,0.84]	0.80 [0.77,0.82]	0.906
	Jaccard Index	0.55 [0.48,0.62]	0.27 [0.23,0.31]	< 0.001
	False Negative Rate	0.38 [0.30,0.46]	0.05 [0.02,0.09]	< 0.001
	Proportion Missed	0.05 [0.04,0.07]	0.01 [0.00,0.02]	< 0.001
Specific Consensus (GPs)	Sensitivity	0.32 [0.25,0.39]	0.95 [0.91,0.98]	< 0.001
	Specificity	1.00 [0.99,1.00]	0.65 [0.62,0.68]	< 0.001
	Precision (PPV)	0.94 [0.87,1.00]	0.28 [0.24,0.32]	< 0.001
	Negative Predictive Value	0.91 [0.89,0.93]	0.99 [0.98,1.00]	< 0.001
	Positive Likelihood Ratio	111.15 [46.58,∞]	2.71 [2.46,3.01]	< 0.001
	Negative Likelihood Ratio	0.68 [0.61,0.76]	0.08 [0.03,0.14]	< 0.001
	Balanced Accuracy	0.66 [0.62,0.69]	0.80 [0.77,0.82]	< 0.001
	Jaccard Index	0.31 [0.24,0.38]	0.27 [0.23,0.31]	0.392
	False Negative Rate	0.68 [0.61,0.75]	0.05 [0.02,0.09]	< 0.001
	Proportion Missed	0.09 [0.07,0.11]	0.01 [0.00,0.02]	< 0.001
Sensitive consensus (GPs)	Sensitivity	0.90 [0.85,0.95]	0.95 [0.91,0.98]	0.074
	Specificity	0.89 [0.87,0.91]	0.65 [0.62,0.68]	< 0.001
	Precision (PPV)	0.53 [0.47,0.59]	0.28 [0.24,0.32]	< 0.001
	Negative Predictive Value	0.98 [0.98,0.99]	0.99 [0.98,1.00]	0.364
	Positive Likelihood Ratio	7.93 [6.72,9.64]	2.71 [2.46,3.01]	< 0.001
	Negative Likelihood Ratio	0.11 [0.06,0.17]	0.08 [0.03,0.14]	0.364
	Balanced Accuracy	0.89 [0.87,0.92]	0.80 [0.77,0.82]	< 0.001
	Jaccard Index	0.50 [0.44,0.56]	0.27 [0.23,0.31]	< 0.001
	False Negative Rate	0.10 [0.05,0.15]	0.05 [0.02,0.09]	0.074
	Proportion Missed	0.02 [0.01,0.02]	0.01 [0.00,0.02]	0.364

**Fig. 2:**
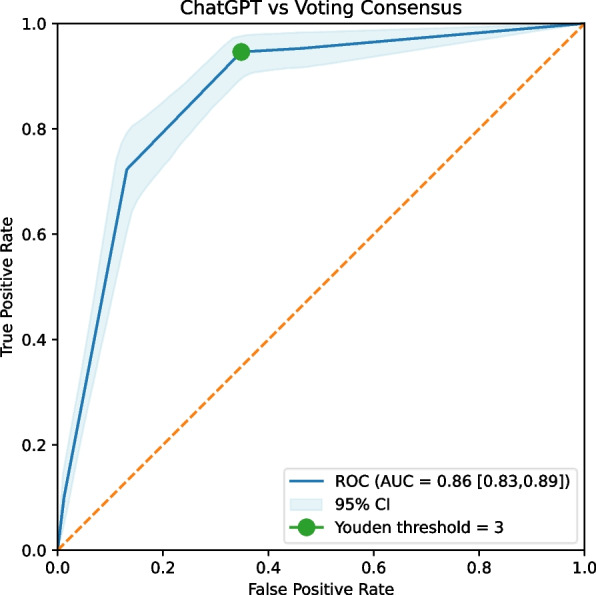
ChatGPT ratings ROC curve. Voting Consensus refers to the final verdict of the expert panel. *ROC* receiver operating characteristics, *AUC* area under the curve, *CI* confidence interval

ChatGPT was asked to rate the citations on a scale from 1 to 5, in alignment with the provided PICOS. The ROC curve derived from this rating process resulted in an AUC of 0.86 [0.83–0.89] (Fig. 2). Based on Youden’s index, the optimal rating threshold for ChatGPT was determined to be ≥ 3 (including ratings 3, 4, and 5 while excluding ratings 1 and 2). Hereafter, all of the reported results are obtained using this threshold unless otherwise noted. ChatGPT achieved a sensitivity of 95% and an NPV of 99%, slightly exceeding the GPs’ sensitive consensus, albeit not statistically significant. However, it did not perform as well in terms of specificity and PPV (Table 3). On the other hand, the AI exhibited remarkably low false negative counts, with only 7 and 8 at thresholds ≥ 2 and ≥ 3, respectively. These are lower than any other rater as shown in Table 4.

**Table 4:** Classification details at different cut-offs over the whole dataset

Rater	Threshold	TPs [95% CI]	TNs [95% CI]	FPs [95% CI]	FNs [95% CI]
ChatGPT	≥ 2	141 [120–163]	564 [531–599]	486 [452–520]	7 [3–12]
	≥ 3*	140 [119–162]	684 [650–718]	366 [333–398]	8 [3–14]
	≥ 4	107 [87–127]	912 [884–938]	138 [116–158]	41 [29–53]
	≥ 5	15 [8–23]	1037 [1014–1060]	13 [6–20]	133 [113–156]
GP 1	n/a	82 [67–99]	990 [965–1013]	60 [46–74]	66 [51–83]
GP 2	n/a	81 [65–99]	1037 [1013–1059]	13 [6–21]	67 [52–84]
GP 3	n/a	109 [90–128]	982 [955–1006]	68 [53–83]	39 [28–52]
Voting consensus	n/a	92 [75–110]	1031 [1007–1054]	19 [11–28]	56 [42–71]
Specific consensus	n/a	47 [34–59]	1047 [1024–1069]	3 [0–7]	101 [83–122]
Sensitive consensus	n/a	133 [113–154]	931 [902–958]	119 [99–139]	15 [8–22]

*Youden’s threshold

*TPs* true positives, *TNs* true negatives, *FPs* false positives, *FNs* false negatives, *CI* confidence interval, *n/a* not applicable

**Fig. 3:**
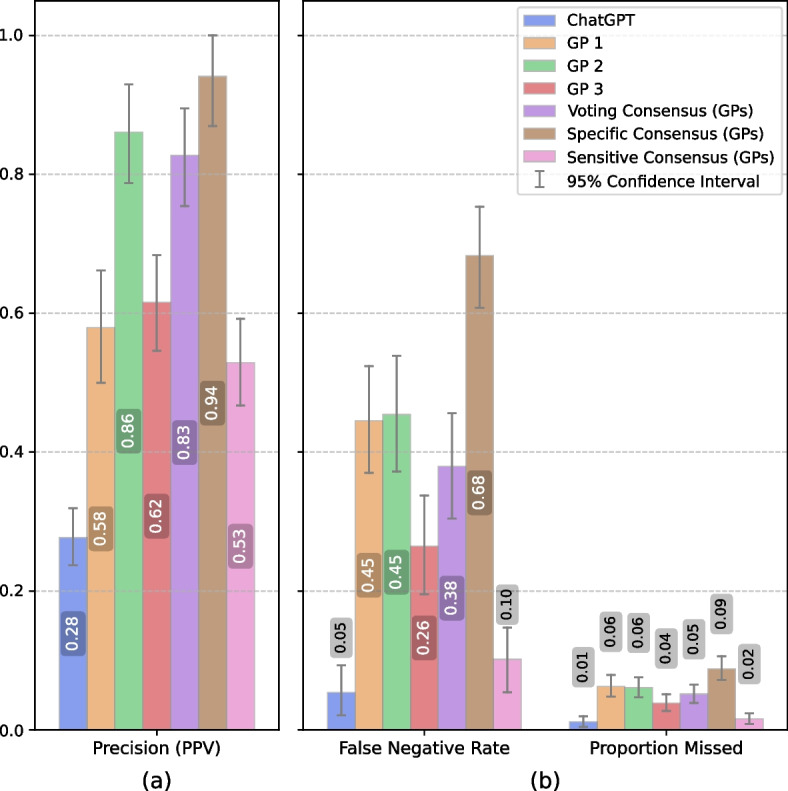
Comparing precision, false negative rate, and proportion missed between raters. Error bars indicate 95% confidence intervals

**Fig. 4:**
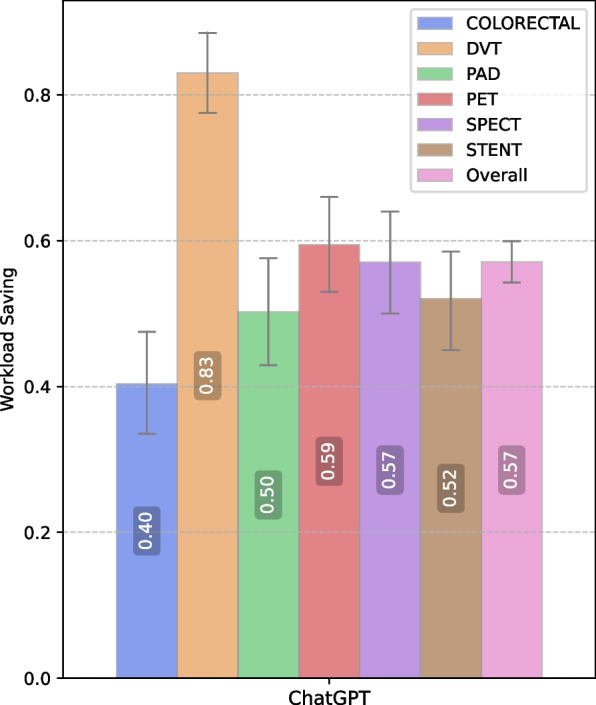
ChatGPT workload savings across topics. Error bars indicate 95% confidence intervals

ChatGPT in general had better performance in terms of false negative rates and proportions missed compared to other raters (both consensuses and individuals) as shown in Fig. 3. Workload savings were especially high, ranging from 40 to 83%, and overall exceeding 50% as depicted in Fig. 4. In addition, it was on average ~ 21 times faster than the physicians’ group.

## Discussion

Three GPs and two experts independently reviewed 1,198 records and categorized them as included or excluded, which took several days. In contrast, it took ChatGPT less than one hour to do the same. ChatGPT exhibited remarkable sensitivity and NPV, both exceeding 95%. Additionally, it had the lowest false negative rates among the raters. On average, the proposed method achieved over 50% workload savings while being an order of magnitude faster.

This study’s primary objective was to assess the efficacy of ChatGPT as an AI adjunct in the task of abstract screening, a time-consuming initial phase of developing systematic reviews and meta-analyses. Rather than complete replacement, the goal is to augment the procedure of human evaluation by reducing workload and increasing efficiency. This can potentially help reduce biases and oversights encountered in the screening process by eliminating subjective inconsistencies and judgmental errors.

While a high AUC suggests a decent concordance between ChatGPT’s ratings and the gold standard, this alone does not translate into lower false negative rates or proportions missed. The count of false negatives, reflected in the aforementioned metrics, is of higher priority in the context of screening. Under specific thresholds (≥ 2 and ≥ 3), ChatGPT exhibited superior performance, achieving very low false negative rates (5%) and proportions missed (1%), compared to its human counterparts. This suggests that citations with low ratings can be confidently eliminated from screening processes, which make up more than half (on average 57%) of the citations. Capitalizing on the speed and scalability of the AI model, the screening process can be split into distinct stages. The first stage allows for the majority of the articles to be excluded automatically. The second stage, characterized by a higher model threshold (e.g., ≥ 4 or = 5), emphasizes the inclusion of only highly relevant articles. Human raters can then evaluate the indeterminate articles (e.g., articles with ratings between 2 and 4) with greater attentiveness. By adopting this hybrid approach, the burden on human raters can be reduced significantly, leading to greater time efficiency and increased accuracy, potentially comparable to those of experts.

We noticed a surprisingly low agreement score between ChatGPT and the other raters (mean κ = 0.27). This might point to the AI model’s fundamentally different “thinking” process, the subjective nature of this process, or simply be due to the selected threshold. This matter requires further investigations, however, since the LLM is basically an average of its training data –as are most statistical models, this could introduce some levels of objectivity into the field. It is imperative to state that ChatGPT has been “aligned” with human preferences using RLHF. While this has greatly increased the usability of the model for everyday tasks, it does not directly translate to better performance in other domains such as medical fields. Thus, we encourage more research in this less-explored area, either by developing medicine-centered language models or by scrutinizing existing models.

Our study incorporated various consensus types: sensitive, specific, and voting. The purpose of this decision was to evaluate the overall performance of the GPs, with each type emphasizing different aspects. Regarding each consensus type, ChatGPT outperformed all with respect to sensitivity (thresholds ≥ 2 and ≥ 3). However, human raters demonstrated superior specificity and precision (PPV). Making use of each rater’s strengths (ChatGPT being more sensitive and humans being more specific), these findings highlight the need for a hybrid approach that incorporates both humans and AI. If ChatGPT alone is to be used, many records will be included unnecessarily leading to a lengthy process of screening the articles in full text.

There are currently several tools, such as Rayyan, Abstrackr, and Colandr, that share a common objective [26–29]. However, they typically employ machine learning (ML) algorithms to rate the articles and need the users to annotate some citations as relevant, unsure, or irrelevant [30]. Some studies have attempted to evaluate the above tools’ performance but mostly relied on retrospective data from earlier systematic reviews [31, 32]. Furthermore, they generally did not have experts as ground truth and solely compared the algorithms’ performance based on the ratings from a single reviewer group [31, 32]. Due to the above reasons and the fact that our approach does not require users to provide annotations, their results may not fully be in alignment with ours.

Gates, A., Guitard, S., Pillay, J. et al. in their review of three ML tools designed for this purpose, employed two approaches: automated approach, delegating all of the screening process to the tools after a 200-record training and semi-automated approach, complementing the work of a single reviewer [33]. They reported by using Abstrackr, DistillerSR, and RobotAnalyst, respectively, the median proportion missed was 5%, 97%, and 70% for the automated simulation and 1%, 2%, and 2% for the semi-automated simulation. Without the need for prior training, ChatGPT with a 1% proportion missed outperforms their automated and is on par with their semi-automated approach (Fig. 3, Table 3).

LLMs are inherently more robust than ML models since they operate excellently even without needing to be pretrained or fine-tuned on a specific dataset (zero-shot performance), as is the case with a lot of ML models [30]. Our research provides a preliminary exploration of the application of LLMs, specifically ChatGPT, in medical research synthesis. The potential for AI models, especially LLMs, could extend to data extraction, objective quality assessment, questionnaire design, and criticism of methods, among other facets of research.

### Limitations

Despite our best efforts to simulate real-world topics, certain constraints limit the broader applicability of our findings.

While our study mainly focuses on the field of radiology, we still had limited resources regarding the number of chosen topics and the diversity of the topics. Limited by time and human resources, we decided to choose 6 titles, each encompassing ~ 200 articles. To cover a representative range of radiology topics, we searched and analyzed the volume of literature in each field to ensure a reasonable distribution. Although we attempted to provide general and representative topics for each field, it is imperative to note that the proposed topics may not entirely reflect real-world issues.

While the PICOS framework remains widely utilized as a prominent approach for defining research scope, alternative frameworks such as SPIDER, SPICE, and ECLIPSE may lead to different outcomes [34–36]. Considering the potential variations in results, further investigations are necessary to comprehensively assess the efficacy and performance of each framework.

Our team of general physicians consisted of three individuals under the age of 30, each with prior experience in article screening and writing review-type pieces. However, they have not had received any specialized training in radiology before the study. To reduce bias, we ensured that they did not communicate with one another and were unaware that they were being compared. It is worth noting that our selection of GPs may not perfectly represent those active in the medical research field, so the generalizability of our results regarding this matter should be verified. Our group of experts, on the other hand, consisted of one young board-certified radiologist and two physicians with over 20 years of experience as radiology researchers with numerous published systematic reviews. To form an even more qualified expert group, we recommend including more experienced radiologists with diverse backgrounds and a higher number of experts.

Although the raters were proficient in English, it was not their native language, thus their performance might have been suboptimal. In contrast, ChatGPT, being an LLM trained on massive English corpora, had an inherent advantage.

While ChatGPT was prompted to rate on a scale of 1 to 5, human raters were tasked with simple inclusion or exclusion. Using an ordinal scale allows us to establish clear cutoffs and prioritize various metrics more effectively. This mismatch could be addressed by asking human raters to provide similar scale-based ratings. However, this would introduce additional complexity and may not mirror real-world practices.

**Fig. 5:**
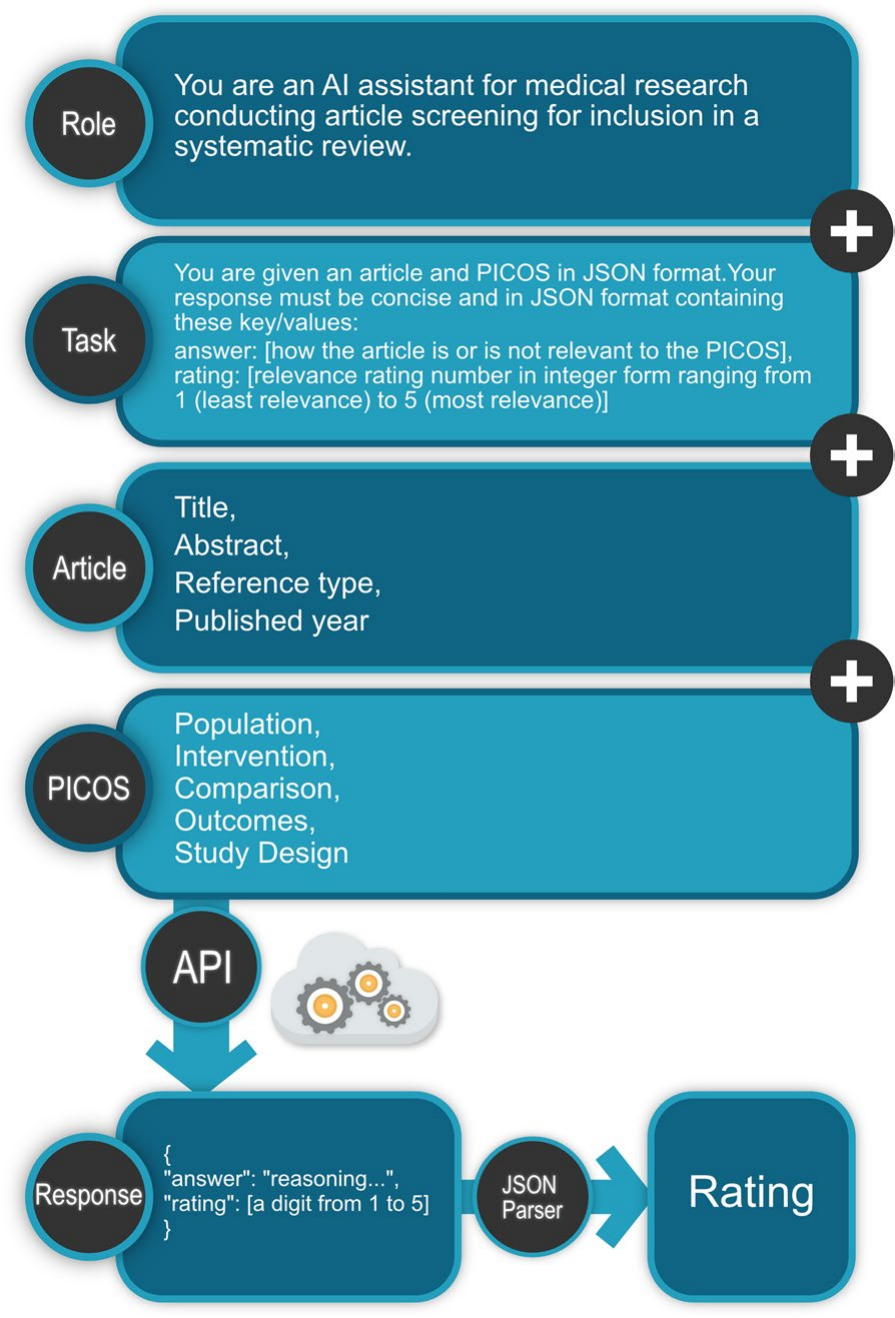
ChatGPT prompting template and rating process. Role: defines the role of AI, or in other words how the AI will behave. Task: the specific task assigned to the AI. Article: provided title, abstract, reference type, and published year. PICOS: determined PICOS based on the specified topic. API: application programming interface, the interface used to communicate with ChatGPT. A temperature of 0.0 was used. Response: the retrieved response from ChatGPT. JSON Parser: a utility tool to parse JSON (JavaScript Object Notation) formatted text. Rating: the rating extracted from the JSON object

Even though our selection of a prompt template was done through trial and error, we only used a single template in the end. There may exist prompt templates with better performance, hence this is an active area of research. A handful of techniques are available for prompting LLMs such as Chain of Thought [37]. These techniques make use of the auto-regressive nature of these models to achieve more accurate responses through “reasoning”. Auto-regressive language models work by predicting the next tokens (could be words or sub-words) in a sequence based on the previous tokens they have already generated or are presented to them (“prompts”) [8]. In this study, we attempted to exploit this feature, similar to the Chain of Thought technique, by having ChatGPT explain its decision before outputting a rating, allowing for more “contemplation” and potentially more reliable responses (Fig. 5).

The optimal threshold determined in this study may not be universally applicable, particularly in cases where the field of study differs. Therefore, the findings of this study call for external validation by other research groups. Moreover, the knowledge gained from our experience can be replicated across diverse medical domains and alternative research methodologies, such as clinical trials, cohort studies, and case–control studies. Evaluating the outcomes in these different fields has the potential to provide a more comprehensive understanding of the overall effectiveness of the approach in this particular context.

Even though we utilized ChatGPT for screening only radiology-centered publications, we believe that the results may very well extrapolate to other fields of medicine, especially fields with more publication volumes such as cardiology and neurology, since these fields most likely constitute a bigger portion of the model’s training data.

For our study, we utilized a particular version of ChatGPT. It is important to note that as the model is continuously updated, other attempts to replicate our study may yield different results. Additionally, analyzing false positive and negative results can inform strategies to further enhance ChatGPT’s efficacy in this regard.

## Conclusion

Our study demonstrates ChatGPT’s potential as a valuable tool in the initial screening phase of systematic reviews confidently excluding more than 50% of irrelevant citations. It showed superior false negative rates and proportions missed within specific thresholds but lagged in specificity and precision (PPV) compared to human raters. A hybrid approach combining AI and human raters could optimize efficiency and accuracy. Further research is necessary to validate findings across fields and explore broader applications of large language models in medical research.

## Declaration of Generative AI and AI-assisted technologies in the writing process

We utilized AI tools, specifically Grammarly GO and ChatGPT, to aid in rephrasing portions of this article. The purpose was to enhance clarity and readability only. Nevertheless, all AI-generated content was meticulously reviewed and verified by the authors.

### Supplementary Information

**Additional file 1.** Further details extending the results and methods of the manuscript.

## Data Availability

The data that support the findings of this study and the screening script are available online. Project name: ChatGPT Screener; Project home page (script): https://github.com/mahbodez/chatgpt_screener; Archived version (containing supporting data): https://doi.org/10.5281/zenodo.10801229; Operating system(s): Platform independent. Programming language: Python. Other requirements: Python installation, required libraries as specified in the repository. License: Creative Commons Attribution-NonCommercial-NoDerivatives 4.0 International License. Any restrictions to use by non-academics: same as license.

## Abbreviations

AI: Artificial intelligence
API: Application programming interface
AUC: Area under the curve
CI: Confidence interval
FNR: False negative rate
GP: General physician
GPT: Generative pretrained transformers
IoU: Intersection over union
LLM: Large language model
ML: Machine learning
NLR: Negative likelihood ratio
NPV: Negative predictive value
PICOS: Population, intervention, comparison, outcomes, study design
PLR: Positive likelihood ratio
PPV: Positive predictive value
RLHF: Reinforcement learning with human feedback
ROC: Receiver-operator characteristics
