# Supplementary material for: Methodological insights into ChatGPT’s screening performance in systematic reviews
Source: BMC Med Res Methodol. 2024 Mar 27;24:78. doi: 10.1186/s12874-024-02203-8 (PMC10976661; doi:10.1186/s12874-024-02203-8)
Supplement: Supplementary file 1 — Additional file 1. Further details extending the results and methods of the manuscript. [file 12874_2024_2203_MOESM1_ESM.docx]

# Supplementary Materials

## Methods

### Data Collection

We searched PubMed for three broad fields of radiology: diagnostic, interventional, and nuclear medicine. Searching “diagnostic radiology”, “interven* radiology”, and “nuclear medicine” yielded 1373836, 160891, and 465747 results respectively. As a result, we distributed six topics among these fields, taking into account their frequency. Subsequently, we designed PICOS (Population, Intervention, Comparison, Outcomes, Study design) for each of the topics. The details regarding search strategies and PICOS are presented in Tables 1 and 2.

The random sampling of the articles was accomplished using a Python script. The script is available at the author’s GitHub repository (<https://github.com/mahbodez/article-sampler>).

## Results

### Inclusion Rates

The details regarding each rater’s inclusion count are available in Table 3.

### Disagreements

Details regarding the distribution of disagreements between the experts are available in Table 4.

### Inter-rater Agreements

Kappa coefficients, categorized by topics, are presented in Figure 1.

### Alternative Gold Standards

In addition to considering the final verdict of the experts as the gold standard, we also employed “sensitive” and “specific” consensuses. ROC curves depicting ChatGPT’s performance while applying different gold standards are presented in Figure 2. Confusion matrices summarizing ChatGPT and general practitioners (GPs) performance across different gold standards and topics are presented in Figures 3 to 9.

Table 1 - Titles and corresponding PICOS

| Title | Population | Intervention | Comparison | Outcomes | Study design |
| --- | --- | --- | --- | --- | --- |
| The diagnostic accuracy of CT colonography in detecting colorectal polyps and cancer: a systematic review and meta-analysis | Patients with suspected or confirmed colorectal polyps and cancer who are eligible for CT colonography. This may include patients with a family history of colorectal cancer, patients with symptoms suggestive of colorectal cancer, or patients who have had an inconclusive or incomplete conventional colonoscopy. | CT colonography, also known as virtual colonoscopy, as a diagnostic tool for detecting colorectal polyps and cancer. This may include the use of different CT colonography techniques (e.g., 2D, 3D, or computer-aided detection) and various bowel preparation methods. | The gold standard for diagnosing colorectal polyps and cancer is typically a conventional colonoscopy or histopathological examination of a biopsy or resected specimens. | The primary outcome of interest would be the diagnostic accuracy of CT colonography in detecting colorectal polyps and cancer. This can be measured using parameters such as sensitivity, specificity, positive predictive value (PPV), negative predictive value (NPV), and the area under the receiver operating characteristic (ROC) curve. Secondary outcomes may include the rate of complications, patient acceptance, cost-effectiveness, and the impact of CT colonography on clinical decision-making. | Randomized controlled trials, prospective or retrospective cohort studies, and diagnostic accuracy studies comparing CT colonography to the gold standard diagnostic tests (e.g., conventional colonoscopy or histopathological examination). |
| Computed tomography angiography versus magnetic resonance angiography for the diagnosis of peripheral arterial disease: a systematic review and meta-analysis | Patients with suspected or confirmed peripheral arterial disease (PAD). This may include patients with symptoms suggestive of PAD (e.g., intermittent claudication, rest pain, or non-healing ulcers) or those with known risk factors (e.g., diabetes, hypertension, hyperlipidemia, smoking, or a family history of PAD). | Computed tomography angiography (CTA) as a diagnostic tool for detecting PAD. This may involve various CTA techniques (e.g., multi-detector, dual-energy) and contrast agents. | Magnetic resonance angiography (MRA) as a diagnostic tool for detecting PAD. This may include different MRA techniques (e.g., time-of-flight, phase contrast, contrast-enhanced) and contrast agents. | The primary outcome of interest would be the diagnostic accuracy of CTA compared to MRA in detecting PAD. This can be measured using parameters such as sensitivity, specificity, positive predictive value (PPV), negative predictive value (NPV), and the area under the receiver operating characteristic (ROC) curve. Secondary outcomes may include the rate of complications, patient acceptance, image quality, cost-effectiveness, and the impact of CTA and MRA on clinical decision-making. | Randomized controlled trials, prospective or retrospective cohort studies, and diagnostic accuracy studies comparing CTA and MRA to a reference standard, such as digital subtraction angiography (DSA) or duplex ultrasound. |
| Accuracy of ultrasound in diagnosing deep vein thrombosis: a systematic review and meta-analysis | Patients with suspected or confirmed deep vein thrombosis (DVT). This may include patients with symptoms suggestive of DVT (e.g., leg pain, swelling, or discoloration) or those with known risk factors (e.g., recent surgery, prolonged immobilization, active cancer, or a history of previous DVT). | Ultrasound, specifically compression ultrasound (CUS) or duplex ultrasound, as a diagnostic tool for detecting DVT. This may involve various ultrasound techniques (e.g., B-mode imaging, color Doppler, or spectral Doppler) and equipment. | The gold standard for diagnosing DVT is typically contrast venography. In this context, the comparator group would consist of patients who underwent contrast venography to confirm the presence or absence of DVT. Alternatively, the comparator could be other noninvasive imaging techniques, such as computed tomography venography (CTV) or magnetic resonance venography (MRV). | The primary outcome of interest would be the diagnostic accuracy of ultrasound in detecting DVT. This can be measured using parameters such as sensitivity, specificity, positive predictive value (PPV), negative predictive value (NPV), and the area under the receiver operating characteristic (ROC) curve. Secondary outcomes may include the rate of complications, patient acceptance, cost-effectiveness, and the impact of ultrasound on clinical decision-making. | Randomized controlled trials, prospective or retrospective cohort studies, and diagnostic accuracy studies comparing ultrasound to the gold standard diagnostic tests (e.g., contrast venography, CTV, or MRV). |
| Comparative effectiveness of drug-eluting stents versus bare metal stents in patients with peripheral artery disease: a meta-analysis | Patients with peripheral artery disease (PAD) who require stent placement to treat arterial stenosis or occlusion. This may include patients with symptoms suggestive of PAD (e.g., intermittent claudication, rest pain, or non-healing ulcers) or those with known risk factors (e.g., diabetes, hypertension, hyperlipidemia, smoking, or a family history of PAD). | Drug-eluting stents (DES) as a treatment option for PAD. These stents are coated with a drug that helps prevent restenosis by inhibiting neointimal hyperplasia. | Bare metal stents (BMS) as a treatment option for PAD. These stents do not have a drug coating and may have a higher risk of restenosis compared to DES. | The primary outcome of interest would be the comparative effectiveness of DES and BMS in terms of clinical outcomes, such as the rate of restenosis, target lesion revascularization, and amputation-free survival. Secondary outcomes may include major adverse cardiovascular events (MACE), stent thrombosis, all-cause mortality, quality of life, and cost-effectiveness. | Randomized controlled trials, prospective or retrospective cohort studies, and registry-based studies. |
| Diagnostic accuracy of PET imaging with 18F-FDG in differentiating malignant from benign adrenal tumors: a systematic review and meta-analysis | Patients with suspected or confirmed adrenal tumors, either discovered incidentally (adrenal incidentalomas) or in the context of known malignancy. This may include patients with symptoms suggestive of adrenal hormone excess or those with known risk factors (e.g., history of cancer, genetic predisposition). | Positron emission tomography (PET) imaging using 18F-fluorodeoxyglucose (18F-FDG) as a diagnostic tool for differentiating malignant from benign adrenal tumors. | The gold standard for diagnosing malignant adrenal tumors is typically a histopathological examination of a biopsy or resected specimens. In this context, the comparator group would consist of patients who underwent histopathological examination to confirm the nature of the adrenal tumor (malignant or benign). Alternatively, the comparator could be other imaging techniques, such as computed tomography (CT), magnetic resonance imaging (MRI), or other PET tracers (e.g., 18F-fluorodopa or 11C-metomidate). | The primary outcome of interest would be the diagnostic accuracy of 18F-FDG PET imaging in differentiating malignant from benign adrenal tumors. This can be measured using parameters such as sensitivity, specificity, positive predictive value (PPV), negative predictive value (NPV), and the area under the receiver operating characteristic (ROC) curve. Secondary outcomes may include patient safety, the impact of 18F-FDG PET imaging on clinical decision-making, and cost-effectiveness. | Prospective or retrospective cohort studies, case-control studies, and diagnostic accuracy studies comparing 18F-FDG PET imaging to the gold standard diagnostic tests (e.g., histopathological examination) or other imaging techniques (e.g., CT, MRI, or other PET tracers). |
| Diagnostic performance of SPECT and SPECT/CT in detecting bone metastases: a systematic review and meta-analysis | Patients with known malignancy and suspected bone metastases. This may include patients with various types of primary cancers, such as breast, prostate, lung, or kidney cancer, and patients with symptoms suggestive of bone metastases (e.g., bone pain, elevated tumor markers, or abnormal findings on other imaging studies). | Single-photon emission computed tomography (SPECT) or SPECT combined with computed tomography (SPECT/CT) as diagnostic tools for detecting bone metastases. This may involve various radiotracers (e.g., 99mTc-MDP, 99mTc-HDP) and equipment. | The gold standard for diagnosing bone metastases is typically a histopathological examination of biopsy specimens. In this context, the comparator group would consist of patients who underwent histopathological examination to confirm the presence or absence of bone metastases. Alternatively, the comparator could be other imaging techniques, such as planar bone scintigraphy, computed tomography (CT), magnetic resonance imaging (MRI), or positron emission tomography (PET). | The primary outcome of interest would be the diagnostic performance of SPECT and SPECT/CT in detecting bone metastases. This can be measured using parameters such as sensitivity, specificity, positive predictive value (PPV), negative predictive value (NPV), and the area under the receiver operating characteristic (ROC) curve. Secondary outcomes may include patient safety, the impact of SPECT and SPECT/CT on clinical decision-making, and cost-effectiveness. | Prospective or retrospective cohort studies, case-control studies, and diagnostic accuracy studies comparing SPECT and SPECT/CT to the gold standard diagnostic tests (e.g., histopathological examination) or other imaging techniques (e.g., planar bone scintigraphy, CT, MRI, or PET). |

Table 2 - Search strategies

| Title | Database | Query | # Of results |
| --- | --- | --- | --- |
| The diagnostic accuracy of CT colonography in detecting colorectal polyps and cancer: a systematic review and meta-analysis | PubMed (MEDLINE) | ("Colorectal"[All Fields] OR "Colon"[All Fields] OR "colonic"[All Fields] OR "Large bowel"[All Fields] OR "Rectal"[All Fields] OR "rectum"[All Fields]) AND ("cancer"[All Fields] OR "carcinom*"[All Fields] OR "neoplas*"[All Fields] OR "malignan*"[All Fields]) AND (("colon*"[All Fields] OR "Intestinal"[All Fields] OR "Rectal"[All Fields] OR "rectum"[All Fields]) AND "polyp*"[All Fields]) AND ("Virtual colonoscopy"[All Fields] OR "Computed tomography colonography"[All Fields] OR "CTC"[All Fields] OR "Computerized tomography colonography"[All Fields]) | 615 |
|  | Embase | ('colorectal' OR 'colon'/exp OR 'colon' OR 'colonic' OR 'large bowel'/exp OR 'large bowel' OR 'rectal' OR 'rectum'/exp OR 'rectum') AND ('cancer'/exp OR 'cancer' OR 'carcinom*' OR 'neoplas*' OR 'malignan*') AND ('colon*' OR 'intestinal' OR 'rectal' OR 'rectum'/exp OR 'rectum') AND 'polyp*' AND ('virtual colonoscopy'/exp OR 'virtual colonoscopy' OR 'computed tomography colonography'/exp OR 'computed tomography colonography' OR 'ctc' OR 'computerized tomography colonography') | 1698 |
|  | Web of Science | ("Colorectal" OR "Colon" OR "colonic" OR "Large bowel" OR "Rectal" OR "rectum") AND ("cancer" OR "carcinom*" OR "neoplas*" OR "malignan*") AND (("colon*" OR "Intestinal" OR "Rectal" OR "rectum") AND "polyp*") AND ("Virtual colonoscopy" OR "Computed tomography colonography" OR "CTC" OR "Computerized tomography colonography") | 950 |
| Computed tomography angiography versus magnetic resonance angiography for the diagnosis of peripheral arterial disease: a systematic review and meta-analysis | PubMed (MEDLINE) | ("Peripheral artery disease"[All Fields] OR "Peripheral vascular disease"[All Fields] OR "extremity arterial disease"[All Fields] OR "Peripheral occlusive arterial disease"[All Fields]) AND ("MRA"[All Fields] OR "MR angiography"[All Fields] OR "Magnetic resonance angiography"[All Fields]) AND ("CTA"[All Fields] OR "CT angiography"[All Fields] OR "Computed tomographic angiography"[All Fields]) | 33 |
|  | Embase | ('peripheral artery disease' OR 'peripheral vascular disease' OR 'extremity arterial disease' OR 'peripheral occlusive arterial disease') AND ('mra' OR 'mr angiography' OR 'magnetic resonance angiography') AND ('cta' OR 'ct angiography' OR 'computed tomographic angiography') | 210 |
|  | Web of Science | ("Peripheral artery disease" OR "Peripheral vascular disease" OR "extremity arterial disease" OR "Peripheral occlusive arterial disease") AND ("MRA" OR "MR angiography" OR "Magnetic resonance angiography") AND ("CTA" OR "CT angiography" OR "Computed tomographic angiography") | 64 |
| Accuracy of ultrasound in diagnosing deep vein thrombosis: a systematic review and meta-analysis | PubMed (MEDLINE) | ("ultrasonography" OR "sonography" OR "US" OR "Doppler ultrasound" OR "duplex ultrasound") AND ("diagnos*" OR "detect*" OR "identify*" OR "determin*") AND ("deep vein thrombosis" OR "DVT" OR "venous thrombosis" OR "deep venous thrombosis") | 7772 |
|  | Embase | ('ultrasonography' OR 'sonography' OR 'us' OR 'doppler ultrasound' OR 'duplex ultrasound') AND ('diagnos*' OR 'detect*' OR 'identify*' OR 'determin*') AND ('deep vein thrombosis' OR 'dvt' OR 'venous thrombosis' OR 'deep venous thrombosis') | 9727 |
|  | Web of Science | ("ultrasonography" OR "sonography" OR "US" OR "Doppler ultrasound" OR "duplex ultrasound") AND ("diagnos*" OR "detect*" OR "identify*" OR "determin*") AND ("deep vein thrombosis" OR "DVT" OR "venous thrombosis" OR "deep venous thrombosis") | 4303 |
| Comparative effectiveness of drug-eluting stents versus bare metal stents in patients with peripheral artery disease: a meta-analysis | PubMed (MEDLINE) | ("drug-eluting stent" OR "DES" OR "drug-coated stent" OR "drug-eluting coronary stent") AND ("bare metal stent" OR "BMS" OR "bare-metal stent" OR "uncoated stent" OR "bare stent") AND ("peripheral artery disease" OR "PAD" OR "peripheral arterial disease" OR "peripheral vascular disease" OR "extremity arterial disease" OR "peripheral occlusive arterial disease") | 102 |
|  | Embase | ('drug-eluting stent' OR 'des' OR 'drug-coated stent' OR 'drug-eluting coronary stent') AND ('bare metal stent' OR 'bms' OR 'bare-metal stent' OR 'uncoated stent' OR 'bare stent') AND ('peripheral artery disease' OR 'pad' OR 'peripheral arterial disease' OR 'peripheral vascular disease' OR 'extremity arterial disease' OR 'peripheral occlusive arterial disease') | 586 |
|  | Web of Science | ("drug-eluting stent" OR "DES" OR "drug-coated stent" OR "drug-eluting coronary stent") AND ("bare metal stent" OR "BMS" OR "bare-metal stent" OR "uncoated stent" OR "bare stent") AND ("peripheral artery disease" OR "PAD" OR "peripheral arterial disease" OR "peripheral vascular disease" OR "extremity arterial disease" OR "peripheral occlusive arterial disease") | 80 |
| Diagnostic accuracy of PET imaging with 18F-FDG in differentiating malignant from benign adrenal tumors: a systematic review and meta-analysis | PubMed (MEDLINE) | ("positron emission tomography 18F-FDG" OR "18F-fluorodeoxyglucose PET" OR "18F-FDG PET imaging" OR "FDG-PET" OR "18F-FDG positron emission tomography") AND ("adrenal tumor" OR "adrenal mass" OR "adrenal neoplas*" OR "adrenal lesion" OR "adrenocortical tumor" OR "adrenal gland tumor") | 115 |
|  | Embase | ('positron emission tomography 18f-fdg' OR '18f-fluorodeoxyglucose pet' OR '18f-fdg pet imaging' OR 'fdg-pet' OR '18f-fdg positron emission tomography') AND ('adrenal tumor'/exp OR 'adrenal tumor' OR 'adrenal mass'/exp OR 'adrenal mass' OR 'adrenal neoplas*' OR 'adrenal lesion'/exp OR 'adrenal lesion' OR 'adrenocortical tumor'/exp OR 'adrenocortical tumor' OR 'adrenal gland tumor'/exp OR 'adrenal gland tumor') | 600 |
|  | Web of Science | ("positron emission tomography 18F-FDG" OR "18F-fluorodeoxyglucose PET" OR "18F-FDG PET imaging" OR "FDG-PET" OR "18F-FDG positron emission tomography") AND ("adrenal tumor" OR "adrenal mass" OR "adrenal neoplas*" OR "adrenal lesion" OR "adrenocortical tumor" OR "adrenal gland tumor") | 116 |
| Diagnostic performance of SPECT and SPECT/CT in detecting bone metastases: a systematic review and meta-analysis | PubMed (MEDLINE) | ("SPECT" OR "single-photon emission computed tomography" OR "single photon emission computed tomography/CT" OR "SPECT-computed tomography" OR "SPECT-CT" OR "hybrid SPECT/CT") AND ("bone metastases" OR "bone metastasis" OR "skeletal metastases" OR "bony metastases" OR "metastatic bone disease" OR "skeletal metastasis") | 385 |
|  | Embase | ('spect' OR 'single-photon emission computed tomography' OR 'single photon emission computed tomography/ct' OR 'spect-computed tomography' OR 'spect-ct' OR 'hybrid spect/ct') AND ('bone metastases' OR 'bone metastasis' OR 'skeletal metastases' OR 'bony metastases' OR 'metastatic bone disease' OR 'skeletal metastasis') | 1619 |
|  | Web of Science | ("SPECT" OR "single-photon emission computed tomography" OR "single photon emission computed tomography/CT" OR "SPECT-computed tomography" OR "SPECT-CT" OR "hybrid SPECT/CT") AND ("bone metastases" OR "bone metastasis" OR "skeletal metastases" OR "bony metastases" OR "metastatic bone disease" OR "skeletal metastasis") | 529 |

Table 3 - Inclusion rates per individual and topic

| Rater | Topic | Per Topic Inclusions | Average Inclusions |
| --- | --- | --- | --- |
| GP 1 | Colorectal | 15.5% | 11.9% |
|  | DVT | 8.5% |  |
|  | PAD | 9.1% |  |
|  | PET | 12.0% |  |
|  | SPECT | 9.5% |  |
|  | Stent | 16.5% |  |
| GP 2 | Colorectal | 13.0% | 7.9% |
|  | DVT | 3.5% |  |
|  | PAD | 4.0% |  |
|  | PET | 9.0% |  |
|  | SPECT | 10.0% |  |
|  | Stent | 7.5% |  |
| GP 3 | Colorectal | 35.0% | 14.8% |
|  | DVT | 5.0% |  |
|  | PAD | 7.1% |  |
|  | PET | 16.0% |  |
|  | SPECT | 14.0% |  |
|  | Stent | 11.5% |  |
| Expert 1 | Colorectal | 23.0% | 12.4% |
|  | DVT | 3.0% |  |
|  | PAD | 5.1% |  |
|  | PET | 18.5% |  |
|  | SPECT | 12.0% |  |
|  | Stent | 12.5% |  |
| Expert 2 | Colorectal | 23.0% | 12.3% |
|  | DVT | 2.5% |  |
|  | PAD | 6.6% |  |
|  | PET | 18.5% |  |
|  | SPECT | 15.0% |  |
|  | Stent | 8.0% |  |

Table 4 - Disagreement distribution between the experts

| **Topic** | **Number of disagreements** | **%** |
| --- | --- | --- |
| **Colorectal** | 10 | 5% |
| **DVT** | 3 | 2% |
| **PAD** | 3 | 2% |
| **PET** | 8 | 4% |
| **SPECT** | 18 | 9% |
| **Stent** | 13 | 7% |

Figure 1 - Kappa coefficients (κ) among raters, subcategorized by topics


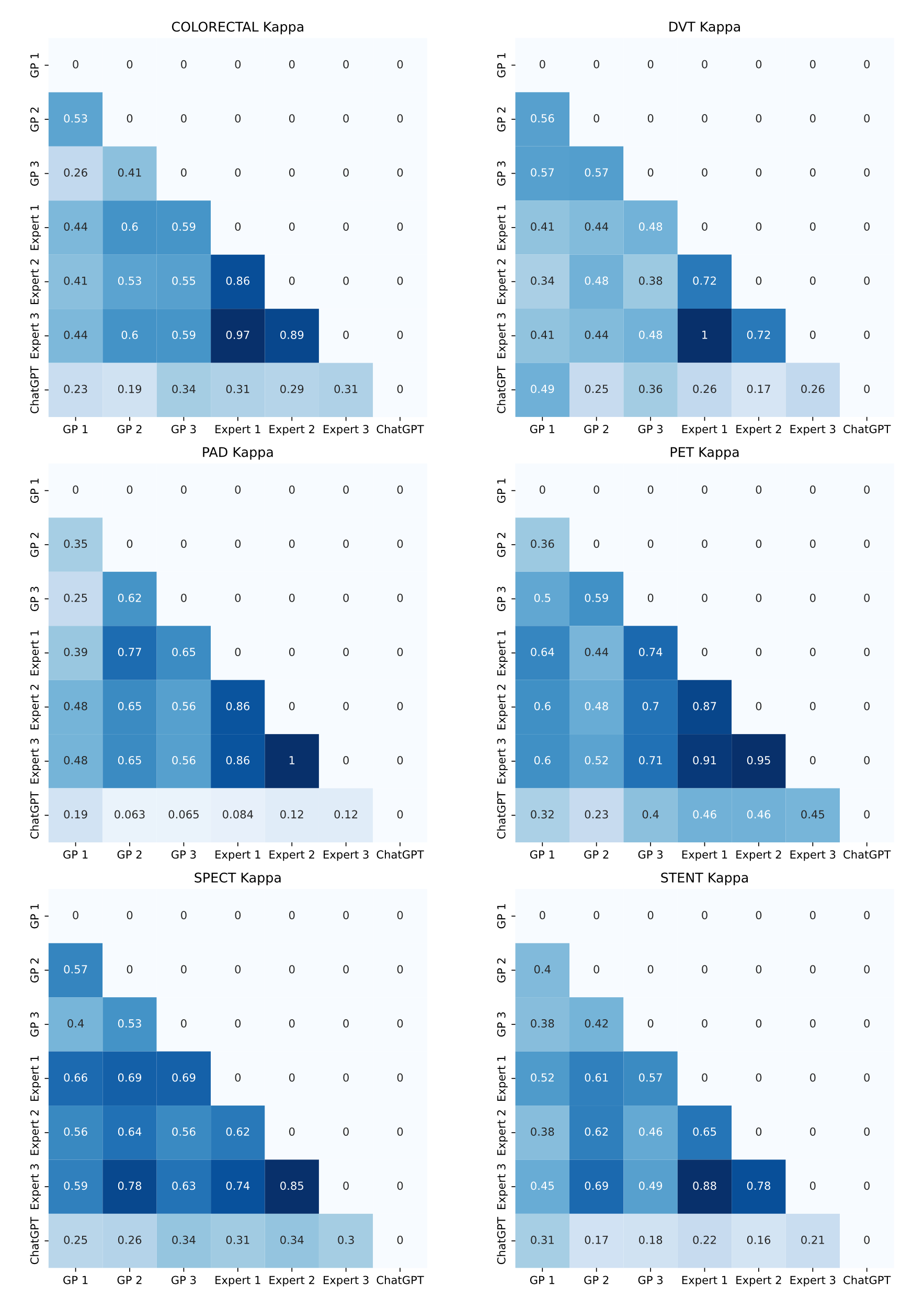


Kappa coefficient matrices, categorized by article topics.

Figure 2 - ROC curves regarding different gold standards


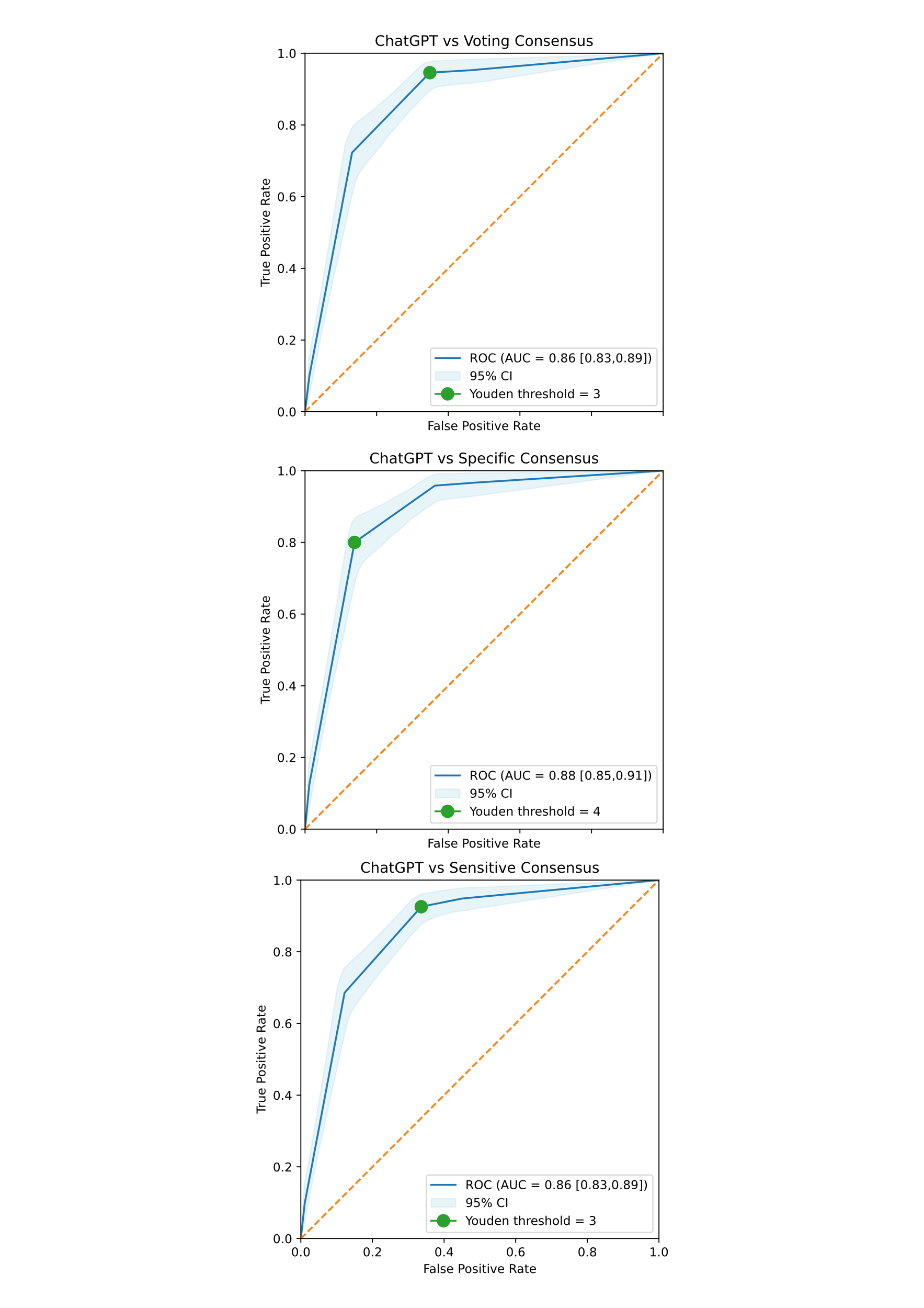


Receiver Operating Characteristic curves (ROCs) corresponding to ChatGPT ratings compared against different gold standards.
Voting Consensus: the final verdict reached by the experts: if the first two experts agree on inclusion/exclusion, the article is included/excluded. Else, the third expert reviews the case and decides on inclusion/exclusion.
Sensitive Consensus: if at least one expert includes the article, then it is included.
Specific Consensus: if both experts include the article, then it is included. Otherwise, it is excluded.
AUC: Area Under the Curve, C-statistic, or C-index. It is used as a measure of concordance between the score and the outcome.

Figure 3 - Overall ChatGPT and GPs’ performance, along with different gold standards


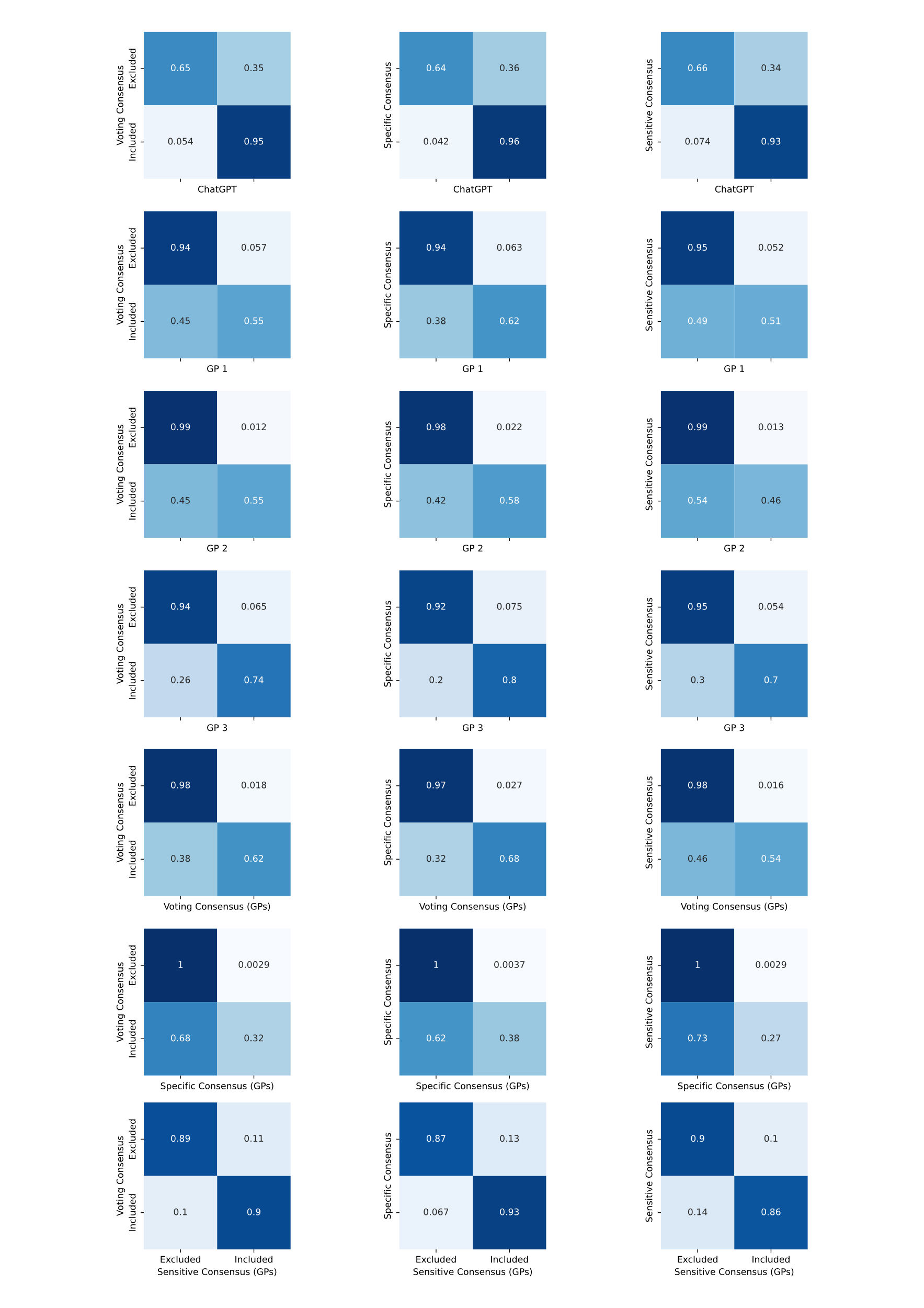


Comparing different raters to each other and ChatGPT, across three different gold standards. The left, middle, and right columns respectively correspond to voting, specific, and sensitive consensuses.

Figure 4 – ChatGPT and GPs’ performance, topic: Colorectal


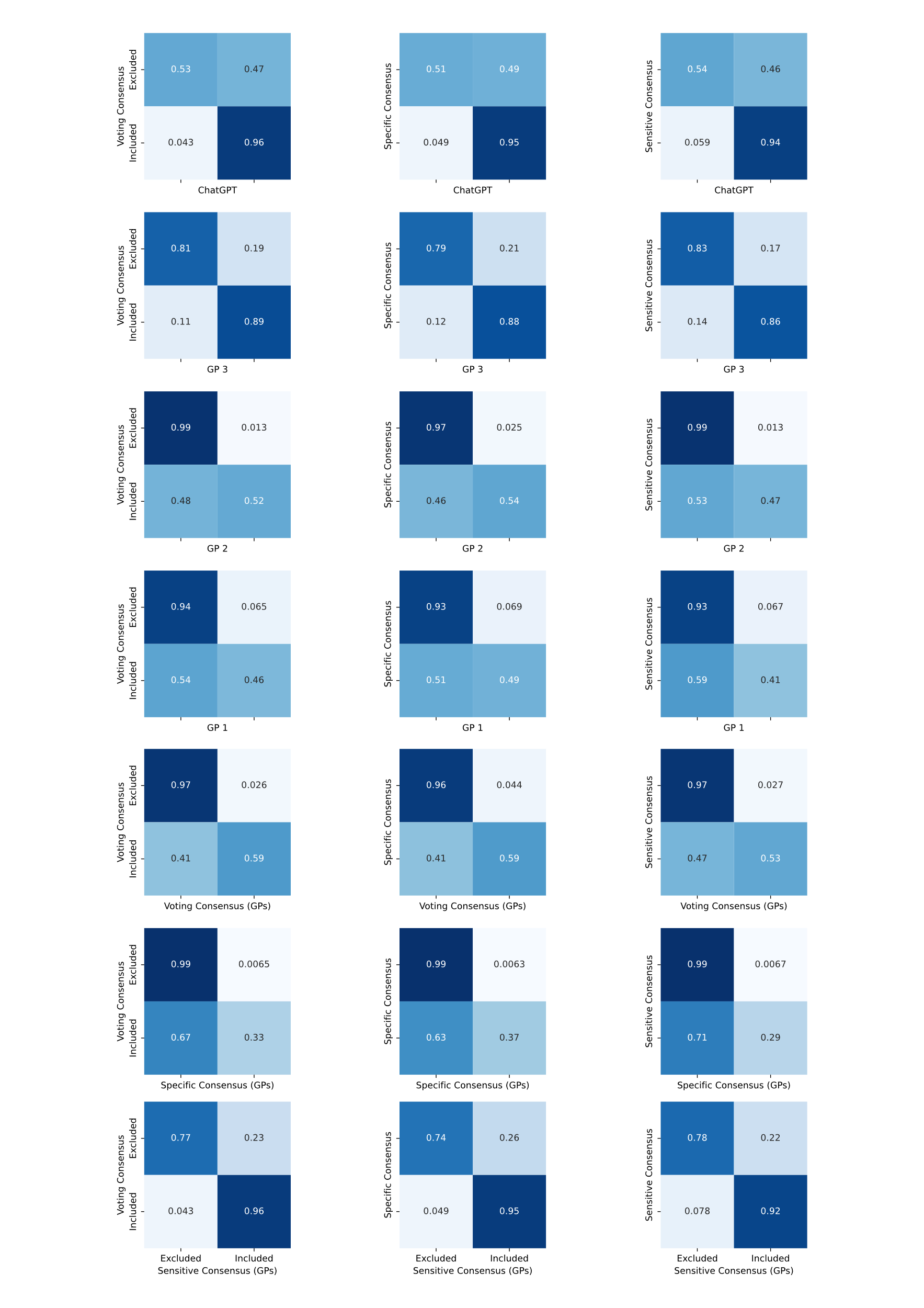


Comparing different raters to each other and ChatGPT, across three different gold standards, regarding the Colorectal topic. The left, middle, and right columns respectively correspond to voting, specific, and sensitive consensuses.

Figure 5 - ChatGPT and GPs’ performance, topic: DVT


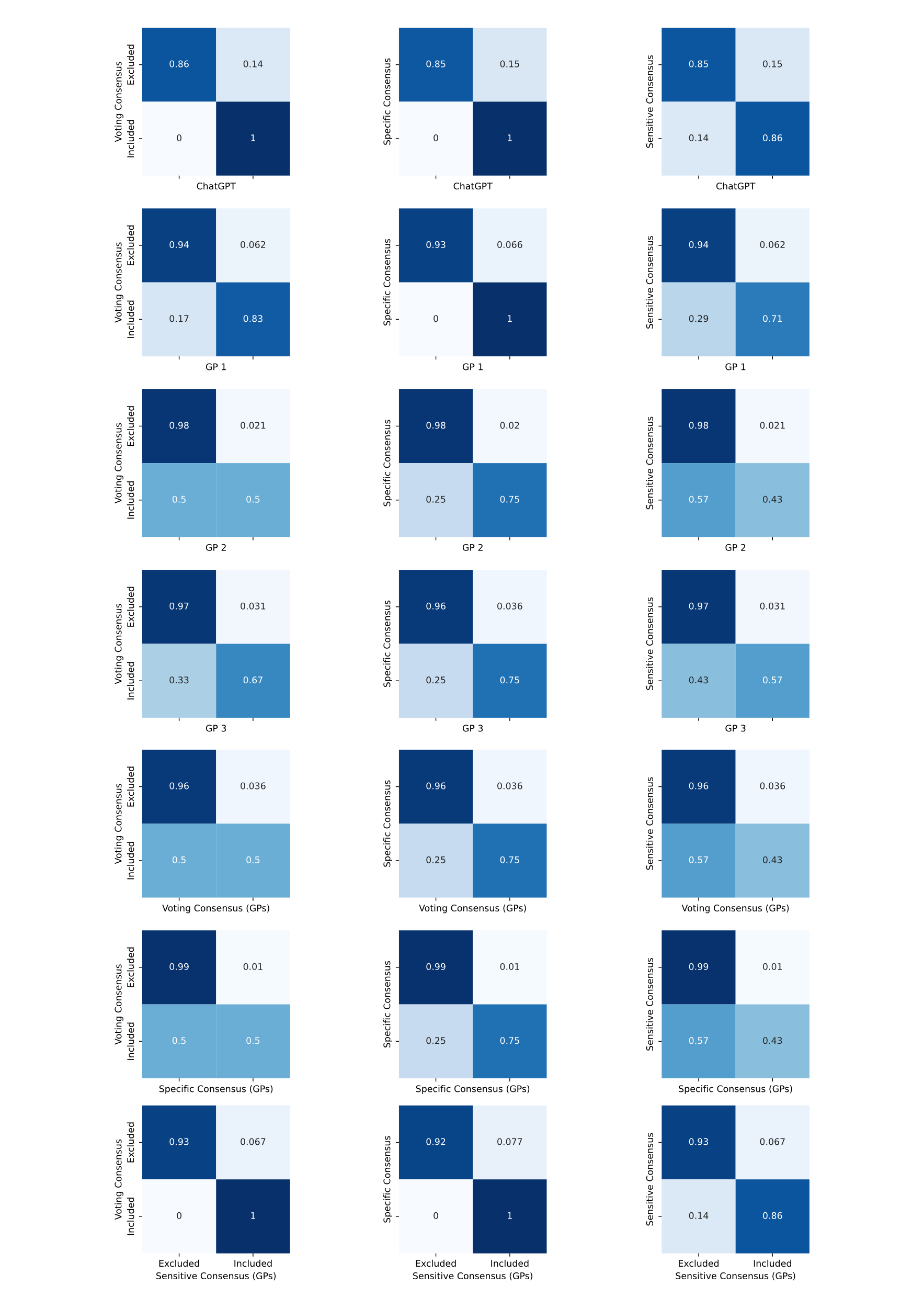


Comparing different raters to each other and ChatGPT, across three different gold standards, regarding the DVT topic. The left, middle, and right columns respectively correspond to voting, specific, and sensitive consensuses.

Figure 6 - ChatGPT and GPs’ performance, topic: PAD


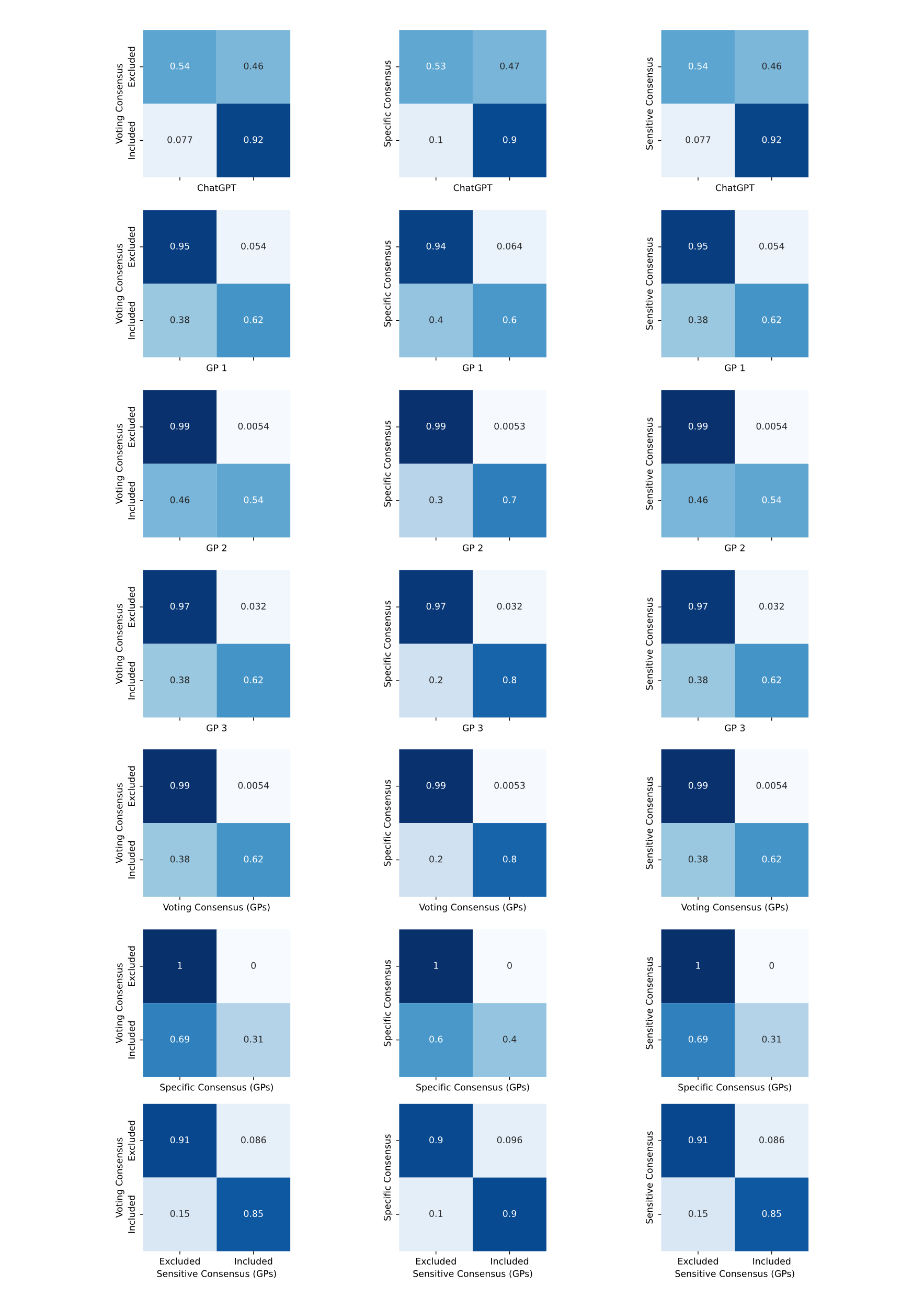


Comparing different raters to each other and ChatGPT, across three different gold standards, regarding the Colorectal topic. The left, middle, and right columns respectively correspond to voting, specific, and sensitive consensuses.

Figure 7 - ChatGPT and GPs’ performance, topic: PET


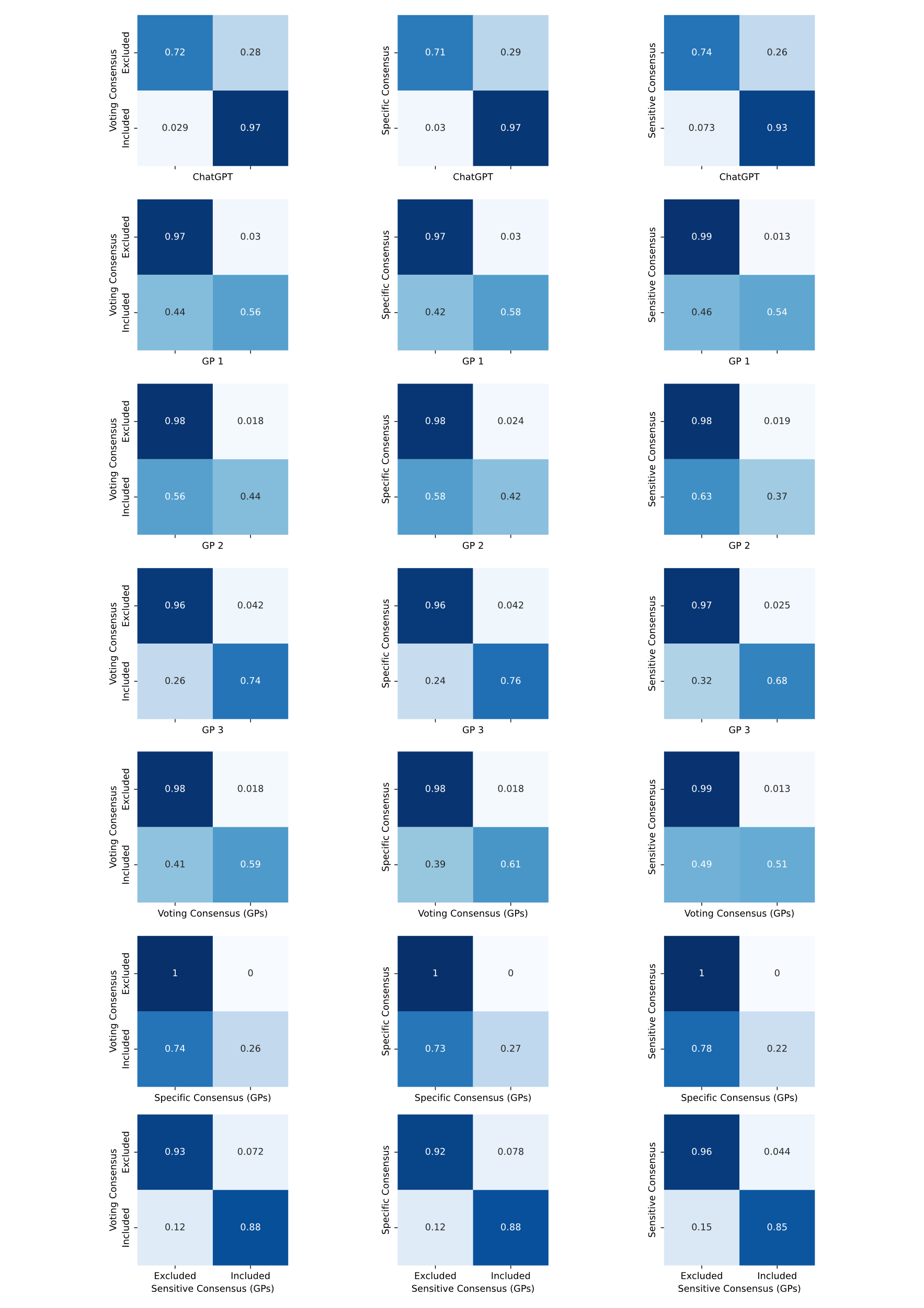


Comparing different raters to each other and ChatGPT, across three different gold standards, regarding the PET topic. The left, middle, and right columns respectively correspond to voting, specific, and sensitive consensuses.

Figure 8 - ChatGPT and GPs’ performance, topic: SPECT


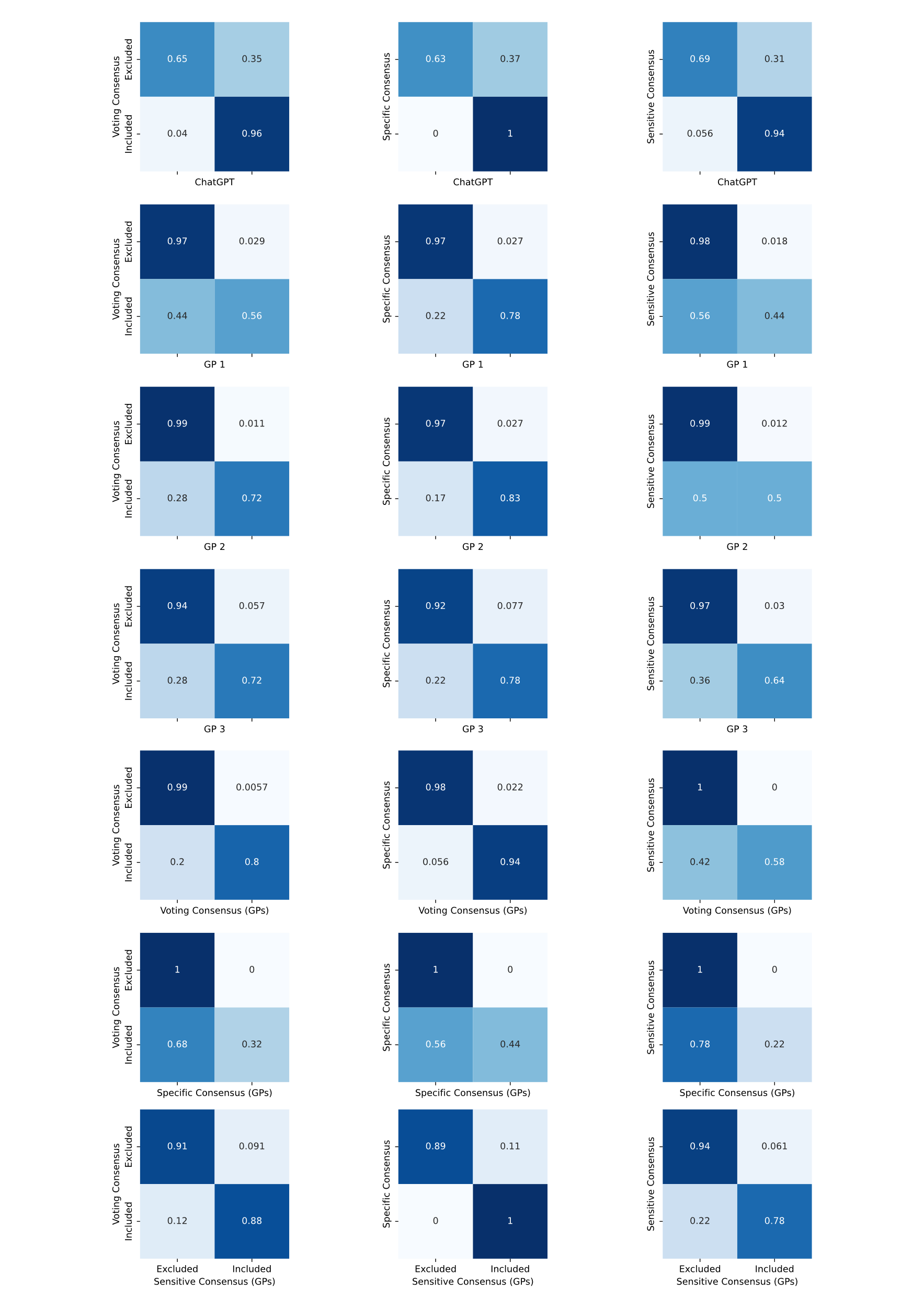


Comparing different raters to each other and ChatGPT, across three different gold standards, regarding the SPECT topic. The left, middle, and right columns respectively correspond to voting, specific, and sensitive consensuses.

Figure 9 - ChatGPT and GPs’ performance, topic: Stent


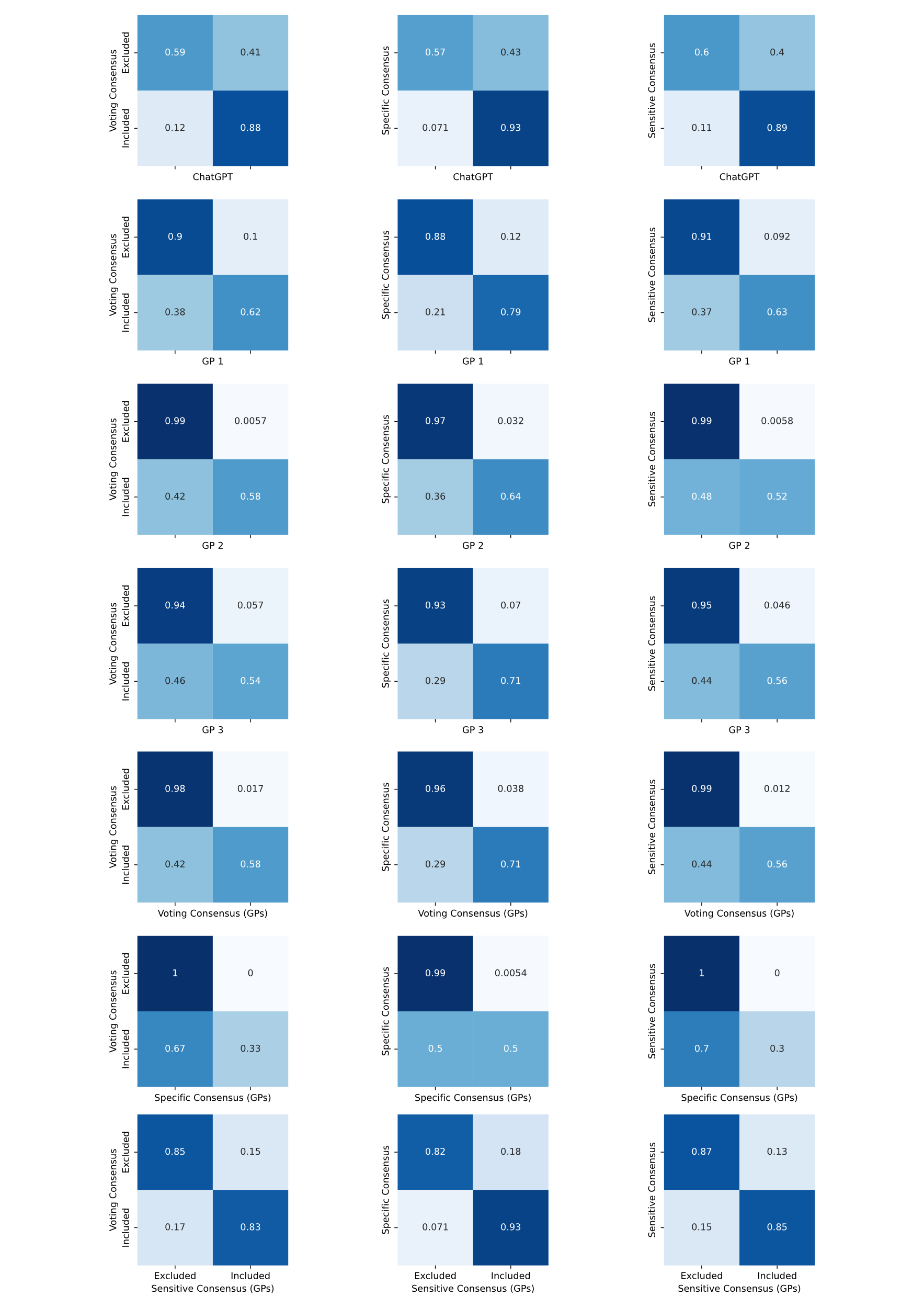


Comparing different raters to each other and ChatGPT, across three different gold standards, regarding the Stent topic. The left, middle, and right columns respectively correspond to voting, specific, and sensitive consensuses.
